# A Scoping Review Protocol: Parenting Experiences and Family Dynamics in Pediatric Burn Care Settings from Hospitalization to the Return Home

**DOI:** 10.3390/nursrep15020071

**Published:** 2025-02-17

**Authors:** Elisabete Cioga, Dulce Cruz, Carlos Laranjeira

**Affiliations:** 1 School of Health Sciences, Polytechnic University of Leiria, Campus 2, Morro do Lena, Alto do Vieiro, Apartado 4137, 2411-901 Leiria, Portugal; 2 Centre for Innovative Care and Health Technology (ciTechCare), Polytechnic University of Leiria, Campus 5, Rua das Olhalvas, 2414-016 Leiria, Portugal; 3 Comprehensive Health Research Centre (CHRC), University of Évora, 7000-801 Évora, Portugal; dcruz@uevora.pt; 4 Department of Nursing, University of Évora, 7000-811 Évora, Portugal

**Keywords:** burned children, experiences, trauma, parents, family dynamics, nursing

## Abstract

**Background:** Evidence indicates that pediatric burns are a significant form of trauma. They affect not only children but also their parents, who often experience short- and long-term psychopathological symptoms. The body of knowledge on the impact of hospitalization on parents has expanded; however, there is a dearth of evidence on the dynamics of parental relationships, parental experiences, and how these experiences affect their parenting. **Objectives:** This study aims to map and summarize the available literature on the repercussions of trauma associated with pediatric burns and hospitalization on parental and family dynamics. **Methods:** A scoping review will be carried out in accordance with the JBI methodology, based on the PCC. Studies involving hospitalized children (up to the age of 18) who have suffered accidental burns and their parents or caregivers will be included. The literature study will examine the effects of pediatric burn-related trauma on family and parental dynamics, emphasizing interventions and adjustment strategies that support children and families affected by this injury. Studies related to hospital settings and returning home will be included and analyzed by two independent reviewers using a standardized form developed for this study. The databases consulted will be Academic Search Complete, Cumulative Index to Nursing and Allied Health Literature (CINAHL), PubMed/Medline, Collection of Psychology and Behavioral Sciences (via EB-SCO), PsycInfo, Cochrane Library, Embase, and Web of Science (Clarivate). **Results:** The results will be summarized narratively, presented in tables or diagrams, to highlight key findings related to parental experiences with burned children, the trauma associated with this episode, and its impact on parenting. In addition, strategies developed within the framework of the care partnership will be highlighted. **Conclusions:** Understanding how family dynamics change after a child suffers a burn injury and goes through the hospitalization process is crucial for nurses to improve their practice. We hope that this review will promote partnership-oriented, family-centered nursing practice in the care of child burn victims and their families, as well as assist in the identification of knowledge gaps in the literature and potential areas for future research and development.

## 1. Introduction

Burns are the fifth-most prevalent kind of trauma globally and constitute a primary cause of mortality and disability in underdeveloped nations [1]. In Europe, a large proportion of pediatric burn cases involve children under the age of five, which accounts for between 50% and 80% of cases [2]. The World Health Organization (WHO) reports that burns result in over 180,000 deaths annually, predominantly in low- and middle-income nations [1,3]. The injuries are due to several factors, including personal behaviors, familial conditions, and broader social and regulatory elements [4]. Household scenarios, especially those involving cooking or open flames, have repeatedly been recognized as a critical risk [4]. Moreover, specific cultural customs, insufficient public knowledge, and restricted availability of safety gear exacerbate the dangers [4]. Burns are one of the most serious injuries that can affect human beings, and in children and adolescents, they represent a significant reason for prolonged hospitalization [5]. Burns can lead to severe sequelae and considerable functional, aesthetic, psychological, and social impairments, along with a high death rate, depending on their severity and extent [5,6]. A recent systematic review emphasizes that children with burn injuries are a particularly vulnerable group and that there is a correlation between increased levels of anxiety and symptoms of traumatic stress, as well as a higher prevalence of psychopathology (i.e., post-traumatic stress disorder, depression, anxiety, and sleep disturbances) over an extended time [5,7].

Traditionally, the emphasis of burn aftercare has been on physical recovery. However, new studies have revealed the negative psychological effects experienced by children who suffer burns, even if the wounds are minimal [8]. The reason for this is that burn injuries significantly impact a child’s physical health and profoundly affect the psychological and emotional well-being of both the child and their family [9].

Pediatric injuries affect the whole family and are experienced as a traumatic event [2]. Children with burns may require prolonged hospitalization, significantly impacting the family routine. However, the severity of the injury determines the need for hospitalization, and treatment can often be carried out on an outpatient basis. Regardless of the place of treatment, burns have a profound impact on family dynamics, requiring adaptations and generating various emotions in parents and other family members [5], leading to an inevitable break with previously familiar family life [10]. Due to the traumatic nature of burns, evidence suggests that both the child burn victims and their parents experience psychopathological symptoms in the short and long term after the accident [10]. Family dynamics are influenced by interactions, roles, and relationships between members (i.e., child–parent, parent–parent, parent–sibling and sibling–sibling), affecting emotional well-being, especially in health situations. Safe and supportive families contribute to the child’s development and help parents deal with challenges. In contrast, stressful dynamics generate conflicts and make it difficult to cope with difficult situations [11]. Parental concerns about their child’s clinical evolution, physical sequelae, and return home are shaped by emotional reactions and uncertainties about the future. Moments of stress and uncertainty alter family relationships, affecting communication and mutual support, and require a joint effort to overcome difficulties and rebuild harmony in the family nucleus [12].

Parenting, or bringing up children, is a process that ensures the health and safety of the child while promoting and supporting their physical, emotional, social, spiritual, and cognitive development from infancy to adulthood [11]. Studies have shown that parents are a key element in the rehabilitation and adaptation of children who have suffered burns [5]. Due to their inherent vulnerability, aggravated by the presence of the burn, children depend especially on their parents to meet their physical, emotional, and social needs [13]. Parents in turn often report a decrease in their ability to meet their children’s needs, potentially threatening the child’s physical and psychological adaptation and the family unit’s well-being [13]. This stems from the fact that when parents see their children undergo painful medical procedures, they need to deal with their own feelings and fears while supporting their children, making parenting a major challenge [12,14]. All these procedures generate a great deal of anxiety in parents, which often translates into a decreased ability to take care of their children and effectively meet their needs [15].

Several studies on the experiences of parents of burned children indicate that parental needs do not differ greatly according to cultural background or socioeconomic circumstances [14,16]. For parents, participating in their children’s medical treatments is a distressing experience. This situation is exacerbated by the burden of other tasks, including caregiving for other children or family members and professional obligations, among others [14]. Furthermore, the very episode of a child’s burns and the inherent consequences can affect the parents’ relationship, and therefore the family and caregiving [14].

Women have always assumed the role of primary family caregiver, and this role is still evident in current literature [17]. Studies focus on the role of mothers during hospitalization, where emphasis is placed on their responsibility for their children’s safety and well-being [18]. Due to the many difficulties experienced and the pressures exerted on the family, mothers express tiredness and an overload of responsibility that, for some, persisted years after the burn, due to appointments and treatments. This factor has also been identified as influencing family and child mental health [18].

Parents often claim they modified their perceptions of anxiety-inducing circumstances and their responses to their child’s injury. They also indicate a modification of their expectations for their damaged child, employing distinct parental strategies for injured and uninjured children, and experiencing challenges in establishing boundaries [15,19]. The literature exposes changes in parental behaviors, characterized by excessively protective attitudes and modifications in family structure to accommodate the injured child [19]. Mothers exhibit protective responses towards their offspring, which may subsequently encourage avoidance coping methods [18].

Adjustments in raising children have an impact on family function and dynamics. Certain changes can increase family cohesion and flexibility after an accident, but others can induce stress, such as conflicts between siblings resulting from unequal treatment [15]. Almost all parents, including those who were not present at the time of the accident, express a feeling of guilt when approached by the authors of various studies [2,5,6,10,12,13,18]. Thus, understanding the meaning that parents attribute to having a child who has suffered a burn injury enriches the professionals who care for the child and their family [5]. According to Bakker et al. [10], pediatric burns cause significant stress and trauma for both the child and the parents, affecting family dynamics and overall well-being. These parents have various demands and concerns throughout the long treatment and recovery process, making comprehensive support crucial for both their well-being and the child’s speedy recovery [8,14]. Emotional support, open communication, and education about post-burn care are essential for recovery and family adjustment [8]. In this context, it is crucial to prepare the family for post-trauma recovery by finding healthy coping mechanisms and encouraging emotional expression after a painful incident [18,20]. The feelings of despair, guilt, and helplessness faced by parents require continuous emotional and psychological support [5]. The implementation of child- and family-centered care, along with parental empowerment, is essential to strengthen families’ ability to cope with trauma [21]. Comprehensive support, including emotional support, detailed information about treatment, and ongoing care, can promote children’s recovery and family adaptation [2]. The implementation of child- and family-centered nursing care [22], along with parental empowerment [23], is essential for strengthening families’ ability to cope with trauma. These models promote an active partnership between parents and nurses, providing emotional, informational, and social support, as well as increasing caregivers’ confidence in their roles.

Considering the influence of parenting on resilience and family well-being, there is a distinct necessity for parental support following a child’s injury to avert possible detrimental effects on family dynamics, particularly when the injured child experiences persistent functional and behavioral impairments, such as those seen in burn victims [15,23]. Understanding the impact on parents has improved, although information regarding parental dynamics during hospitalization and the significance of these events on parenting remains insufficient. Hospitalization inevitably leads to separation from home and disruption of family life, due to the long periods of hospitalization and the numerous invasive procedures required for treatment [23]. At the same time, deep dermal burns cause permanent scarring, which may require lifelong medical follow-up [10]. Thus, despite recent developments, burns have a massive impact on the lives of children and their families.

Burns in children represent a traumatic event that has a significant impact on family dynamics. Although there are studies on clinical management and the emotional impact on caregivers [2,5,7,10,14], there is a lack of research that comprehensively explores family experiences in this context. There is a need for a review that will allow us to understand the experiences of parents with children hospitalized with burns, as well as the associated traumas and how these influence family dynamics. Therefore, this review will aim to: (a) map and summarize the available literature on repercussions of trauma associated with pediatric burns and how the hospitalization process affects parental and family dynamics; (b) identify parental and family adjustment strategies in caring for children who have suffered burns; and (c) characterize the challenges that parents face when preparing for hospital discharge. Hopefully, this review will help identify gaps in evidence and guide future research and effective interventions. A synthesis of the evidence will contribute to developing family-centered nursing interventions, improving support during hospitalization and discharge.

## 2. Materials and Methods

The suggested scoping review will be conducted in accordance with the Preferred Reporting Items for Systematic Reviews and Meta-Analyses for Scoping Reviews (PRISMA-ScR) [24] and the updated JBI methodology for scoping reviews [25,26]. The JBI framework is influenced by the work of Arksey and O’Malley [27], a subsequent expansion of their framework by Levac et al. [28], and additional improvements by Peters et al. [29]. The scoping review involves the following steps: “(1) identifying the research question(s), (2) identifying relevant studies, (3) selecting studies for analysis, (4) documenting and collating the data, and (5) summarizing and reporting the results” [29]. The aforementioned strategies will be implemented in the course of this investigation. The protocol for this review has been prospectively registered with the Open Science Framework (https://doi.org/10.17605/OSF.IO/754ZP, accessed on 5 January 2025). This study does not necessitate ethics approval, as the scoping review methodology involves the review and collection of data from publicly available materials.

### 2.1. Review Questions

The review questions were developed to reflect the general and specific objectives of this scoping review, in order to ensure a comprehensive collection and synthesis of data on the impact of pediatric burns on family and parental dynamics.

What are the reported experiences of parents of children hospitalized with burns?

How does the process of hospitalizing a child with burns affect parental and family dynamics?

What adjustment strategies are used by parents and families in caring for children hospitalized with burns?

What are the main challenges faced by parents of children with burns in preparing for hospital-to-home transitions?

### 2.2. Identifying Relevant Studies

This scoping review will be conducted using the participants–concept–context (PCC) mnemonic proposed by the Joanna Briggs Institute [25,26]. The initial selection criteria will be established accordingly.

#### 2.2.1. Participants

The review will evaluate all studies involving children (up to the age of 18 at the time of the burn) hospitalized (inpatient or outpatient) due to accidental burns (with no minimum or maximum length of stay), as well as their parents or guardians, including a biological or adoptive parent, stepparent, legal guardian, or other individual who has legal custody or responsibility for the child [29]. We will also incorporate studies with single-parent families and cases when parents do not reside together. There will be no limitations on the characteristics or demographic data of the parents. All types of burns will be included. Studies in which the pediatric burn was not accidental will be excluded.

#### 2.2.2. Concept

Literature that examines the influence of trauma associated with pediatric burns and hospitalization on family dynamics and parenting will be examined in relation to the concept. Parenting or child-rearing is a process that ensures the health and safety of a child, promotes and supports the physical, emotional, social, spiritual, and cognitive development of a child from infancy to adulthood, and prepares children for a productive adult existence. Family dynamics are the patterns of interaction among relatives, their roles and relationships, and the various factors that influence their interactions [11].

The central concept of this review covers the literature that explores the influence of trauma associated with pediatric burns and hospitalization on family dynamics and parenting. Parenting is the process of ensuring a child’s health and safety, promoting physical, emotional, social, spiritual, and cognitive development from infancy to adulthood, as well as preparing them for a productive existence [11]. Family dynamics refers to the patterns of interaction between family members, their roles and relationships, and the factors that influence these interactions [11,12]. Trauma in this context refers to the psychological and emotional stress experienced by both the child and their family due to severe burn injuries and subsequent hospitalization [7,8,12,18,30]. Challenges faced by parents include a combination of emotional, logistical, and psychological difficulties [2,5,7,8,9,10,12,13,18,30,31]. The review will seek to understand how burn trauma and subsequent hospitalization affect parents’ psychological well-being, parenting practices, family dynamics, and the adjustment strategies adopted.

#### 2.2.3. Context

This review will consider the literature on hospital and home-based services that care for children and families affected by burns, including pediatric burn units, pediatric intensive care units, general pediatric wards that treat burn cases, and specialized rehabilitation centers for pediatric burns. Studies from diverse cultural and socioeconomic contexts will be considered to allow for a comprehensive understanding of family experiences in different realities. The choice of context is crucial because of its relevance to parental and family adjustment. Hospitalization causes significant changes in routine, the role of parents, and family relationships. Returning home introduces challenges related to ongoing care, the after-effects of burns and the child’s reintegration. Understanding the impact of these periods is essential for mapping parental experiences and proposing family-centered care strategies [30]. All relevant studies will be included to synthesize the existing evidence.

#### 2.2.4. Types of Sources

This scoping review will examine experimental studies and observational research (with descriptive, exploratory, and analytical designs). Qualitative studies (e.g., phenomenological, ethnographic, grounded theory, case studies) and mixed-method studies will also be included. Moreover, systematic reviews and meta-analyses that fulfill the inclusion criteria will be evaluated based on the research question. Government reports, theses, and dissertations will be included to capture evidence not published in academic journals. Editorials, opinion pieces, and conference abstracts will be removed.

### 2.3. Selecting Studies for Analysis

A complete search strategy was drawn up by two members of the research team and an experienced librarian. The search will take place in three stages, seeking to identify published and unpublished studies. The first stage is a restricted preliminary search on Medline (PubMed) and Cumulative Index to Nursing and Allied Health Literature (CINAHL) to locate articles on the subject. The terminology in the titles and abstracts of the relevant articles, together with the index terms (MeSH thesaurus and CINAHL subject headings) employed to describe them, will be used to formulate a comprehensive, second-stage search strategy on the following databases: Academic Search Complete; CINAHL Complete (via EBSCOhost); PubMed/Medline; Psychology and Behavioral Sciences Collection (via EBSCO), PsycInfo, Cochrane Library, Embase, and Web of Science (Clarivate). The pilot search strategy was developed using the PubMed/Medline database (Table 1). The detailed search strategy can be found in Table S1 and was conducted on 17 January 2025. Then, the search strategy will be adapted for each database and/or source of information included. In the last step, the list of references from all the sources of evidence included will be examined for additional studies. Studies published in English, Portuguese, and Spanish (languages spoken by the authors) will be included, with no time frame. Sources of unpublished studies and gray literature will be searched using Scopus, Web of Science, Google Scholar (the first 10 pages of results), ProQuest Dissertations, and Theses Global. It should be noted that the search strategy may be modified during the review process to account for new information.

### 2.4. Study/Source of Evidence Selection

After the search, all the citations found will be compiled and uploaded to Mendeley Desktop, and duplicates will be eliminated. The results will then be exported to Ryyan (http://rayyan.qcri.org) to facilitate study screening and selection. Two independent reviewers will then assess the titles and abstracts according to the review’s inclusion criteria. The same two reviewers will independently assess the full texts of potentially eligible studies. A pilot test of the selection process will be conducted on a sample of 50 citations to ensure consistency between reviewers. All the bibliographical references of the selected articles and studies will be examined in order to identify potential additional studies that could be included in this review. Elimination based on the full text will be documented and presented in the scoping review. Studies will be excluded if they do not meet the inclusion criteria. Disagreements between reviewers at each level of the selection process will be resolved through discussion or with the involvement of additional reviewers for the final decision. In studies with incomplete information, the authors will be contacted for additional information.

The outcomes of the search and the study inclusion process will be comprehensively detailed in the final scoping review and illustrated in a Preferred Reporting Items for Systematic Reviews and Meta-Analyses for Scoping Reviews (PRISMA-ScR) flow diagram [25].

### 2.5. Charting and Collating the Data

Data will be retrieved from the papers included in the scoping review by two independent reviewers utilizing a data extraction tool created by the authors and then compared. Reviewers will receive training on the use of the data extraction form before starting the process. The retrieved data will include details regarding the participants, concept, context, study methodologies, and principal findings pertinent to the review questions. A draft extraction form is included (Table S2). The data extraction form will be tested on a sample of five included studies to ensure its adequacy and consistency. The draft data extraction tool will be amended and refined as required during the data extraction process. The adjustments will be specified in the scoping review. Discrepancies among reviewers will be addressed through dialogue or by involving a third reviewer. Authors of studies will be contacted to seek missing or extra data, if necessary.

### 2.6. Summarizing and Reporting the Results

As suggested by the JBI, the results will be presented in the form of tables, whenever possible, to facilitate the mapping of the data extracted and in a narrative synthesis. The tables will be developed and adjusted over the course of the data extraction, seeking to answer the review question and its objectives, namely, the experiences of parents with burned children, the trauma associated with this episode and parenting, and whenever possible the identification of strategies developed within the care partnership. The information they contain will be supplemented with a narrative synthesis to provide a detailed description and thus a comprehensive understanding of the phenomena studied. The narrative synthesis will be conducted in three stages: (1) organizing studies into logical categories, (2) analyzing findings within these categories, and (3) synthesizing findings across categories to produce an overarching line of argument [32]. The results will be mapped according to the four main research questions, with subsections for emerging themes within each question. Illustrative quotes from included studies will be used to exemplify key themes in the narrative synthesis. The analysis will be conducted by one author (E.C.) and validated by two additional authors (D.C. and C.L.). The results will be disseminated in accordance with PRISMA-ScR reporting standards, following its main elements: (a) clear description of the objectives and research questions; (b) details of the eligibility criteria and search methods; (c) explanation of the study selection and data extraction processes; (d) presentation of the results with narrative syntheses; and (e) discussion of the implications of the findings and identification of gaps in the literature. Adherence to PRISMA-ScR will benefit the review’s quality and transparency by ensuring that all stages are clearly documented and reproducible, thereby facilitating the understanding of the methods employed and strengthening the reliability of the results. The PRISMA-ScR checklist will be used when writing the final report, ensuring that all essential items are adequately addressed. This practice promotes scientific integrity and contributes to the review’s validity and usefulness for various researchers and health professionals. Based on the research by Arksey and O’Malley [27], this scoping review will not assess the methodological quality or rigor of the included studies.

## 3. Results

The review began in January 2025, and the official literature search will be finished by the end of March 2025. Results will be presented in three primary formats: summary tables, visual figures, and a narrative synthesis. A PRISMA flow diagram will be presented for the study selection process. A concept map will be developed to illustrate the relationships among the main themes identified in the review, providing an overview of the findings. The dissemination strategy will involve publishing the review results in a peer-reviewed, open-access healthcare journal and presenting the findings at prestigious scientific conferences. Findings will be disseminated to an academic audience by June 2025.

## 4. Discussion

This scoping review aims to understand how trauma associated with having a child who has been accidentally burned influences parental and family dynamics. Previous studies have focused on the support needs of parents [2] and the importance of understanding parents’ experiences and their psychosocial support needs [11]. There are studies on how parents cope with the stress of their child’s injury during acute hospitalization and how the traumatic nature of pediatric burns affects the family from the caregiver’s point of view [13,18]. The results of these studies indicate the need for further research to explore how the trauma of having a child burn victim influences the parental role and the family [4,10,13,21,30]. As such, based on the evidence, this review will have significant implications for the care of these children and their families, demonstrating the urgent need to adopt a holistic approach. By focusing not only on the physical aspects, such as pain, but also on how the event of having a burned child influences parenting and family dynamics, the research will promote family-centered care [21,23], as it recognizes the interdependence between the child’s well-being and family performance. By carrying out this study and disseminating it, we aim to raise awareness among nurses who care for these families about the importance of the family’s emotional needs. We also hope that mapping the existing literature will lead to future research aimed at improving nursing interventions centered on family dynamics to help improve the lives of these families. The results of this review will be discussed in detail and in alignment with each research question.

Only articles written in English, Portuguese, and Spanish will be considered, which means that other sources that may exist in other languages may not be included. While the inclusion criteria are broad, there is a chance that some relevant sources will not be included. Furthermore, although a stakeholder or expert consultation may enhance the social validity of the research, this approach will not be included in this review. Given that the included studies will not be subjected to critical appraisal, it will not be possible to verify the rigor or reliability of these studies. However, this review can contribute to an overall assessment of the quality of the available research and identify areas that need further investigation.

## 5. Conclusions

Burns have a tremendous impact on the lives of children and their families. This review will aim to map and synthesize the repercussions of trauma associated with pediatric burns and hospitalization on parental dynamics. To this end, an investigation will be carried out into the existing evidence on the experiences of parents with burned children and how these experiences influence parenting and family dynamics. With the development of this protocol, steps are outlined to methodically investigate this topic and provide an opportunity to obtain more information on the orientation of the proposed review.

## Figures and Tables

**Table 1:** Search strategy used on PubMed/Medline for identifying potentially pertinent articles.

PCC	TITLE-ABS-KEY
Population	(“children” OR “chil*” OR “paediatric” OR “pediatric” OR “youth” OR “young” OR “adolesc*”) AND (“burn*” OR “scald*” OR “thermal injury” OR “thermal injuries” OR “burn wound”) AND (“family” OR “relative*” OR “carer*” OR “caregiver” OR “family caregiv*” OR “parent*” OR “guardian”)
Concept	(“parent role” OR “challenge*” OR “need*” OR “demand*” OR “support” OR “experience*” OR “coping” OR “adjustment”)
Context	(“p?ediatrics burn unit” OR “hospital*” OR “burn centre” OR “burn care center” OR “Inpatient burn unit” OR “Intensive care unit” OR “burn care service” OR “hospital discharge” OR “Home care”)

* = Truncation; ? = term variations.

## Data Availability

No new data were created or analyzed in this study. Data sharing is not applicable to this article.

## Supplementary Materials

The following supporting information can be downloaded at https://www.mdpi.com/article/10.3390/nursrep15020071/s1. Table S1: Detailed search strategy for PubMed/Medline. Table S2: Draft data extraction tool.

## Public Involvement Statement

There was no public involvement in any aspect of this research.

## Guidelines and Standards Statement

This manuscript follows the Joanna Briggs Institute (JBI) [24,25] guidelines and the Preferred Reporting Items for Systematic Reviews and Meta-Analyses for Scoping Reviews (PRISMA-ScR) guidelines [23].
