# Supplementary material for: A Scoping Review Protocol: Parenting Experiences and Family Dynamics in Pediatric Burn Care Settings from Hospitalization to the Return Home
Source: Nurs Rep. 2025 Feb 17;15(2):71. doi: 10.3390/nursrep15020071 (PMC11858328; doi:10.3390/nursrep15020071)
Supplement: Supplementary file 1 [file nursrep-15-00071-s001.zip › Table S1. Draft Pubmed search.pdf]

Table S1: Detailed search strategy for PubMed/MEDLINE (The search was conducted on 17 January 2025)

Search	Query[1]	Records retrieved
**#1**	("parents"[MeSH Terms] OR ("famil*"[Title/Abstract] OR "relative*"[Title/Abstract] OR "career*"[Title/Abstract] OR "caregiv*"[Title/Abstract] OR "family care"[Title/Abstract] OR "parent*"[Title/Abstract]) AND ("chil*"[Title/Abstract] OR "p?ediatric"[Title/Abstract] OR "young"[Title/Abstract] OR "youth"[Title/Abstract] OR "adolesc*"[Title/Abstract] OR ("child"[MeSH Terms] OR "child, hospitalized"[MeSH Terms]) AND ("burn*"[Title/Abstract] OR "scald*"[Title/Abstract] OR "thermal injur*"[Title/Abstract] OR "burn wound*"[Title/Abstract] OR "Burns"[MeSH Terms])	1,445
**#2**	("parent role"[Title/Abstract] OR "challenge*"[Title/Abstract] OR "need*"[Title/Abstract] OR "demand*"[Title/Abstract] OR "support*"[Title/Abstract] OR "experience*"[Title/Abstract] OR "perception*"[Title/Abstract] OR "impact"[Title/Abstract] OR "coping"[Title/Abstract] OR "adjust*"[Title/Abstract] OR ("coping skills"[MeSH Terms] OR "adaptation, psychological"[MeSH Terms] OR "psychological adaptation"[Title/Abstract] OR "life change events"[MeSH Terms] OR "life change events"[Title/Abstract])	3,962,410
**#3**	("Hospitals"[MeSH Terms] OR "home care services, hospital based"[MeSH Terms]) AND ("burn unit"[Title/Abstract]) OR "pediatric hospital"[Title/Abstract] OR "burn center*"[Title/Abstract] OR "burn care center*"[Title/Abstract] OR "p?ediatric intensive care unit"[Title/Abstract] OR "intensive care units, pediatric"[Mesh Terms] OR "burn care service"[Title/Abstract] OR "hospital discharge"[Title/Abstract] OR "home care"[Title/Abstract])	410,221
**#4**	#1 AND #2 AND #3	55
**#5 Filters applied**	Language: English, Portuguese, Spanish	50

# = Research line, * = Truncation, ? = term variations.

[1] A similar strategy will be used for the remaining databases.
