# Supplementary material for: A Scoping Review Protocol: Parenting Experiences and Family Dynamics in Pediatric Burn Care Settings from Hospitalization to the Return Home
Source: Nurs Rep. 2025 Feb 17;15(2):71. doi: 10.3390/nursrep15020071 (PMC11858328; doi:10.3390/nursrep15020071)
Supplement: Supplementary file 1 [file nursrep-15-00071-s001.zip › Table S2. Data_Extraction_tool.pdf]

Table S2: Draft Data Extraction Tool

Evidence Source Details and Characteristics	Standardization
Authorship	Detail study authorship
Database	According to origin during the selection process
Year of publication	Year the study was published
Country	Place of origin of the study
Objective	Detail study objective
Type of research and methodological approach	Detail the type and design of research
Study population and sample size	Detail the population and research participants
Impact of hospitalizing a child with a burn on parental and family dynamics	Detail the individual, family and social impact associated with hospitalization in parental and family dynamics
Main repercussions of trauma associated with paediatric burns on parental and family dynamics	Detail the individual, family and social repercussions of trauma associated with paediatric burns on parental and family dynamics
Parental and family adjustment strategies in caring for children hospitalized with burns	Detail the adjustment/coping strategies used by parents in caring for children hospitalized with burns
Challenges parents face in the process of preparing for hospital-to-home transitions	List the major challenges that parents faced in transition to home care
Implications/recommendations for nursing interventions	Identify strengths and weaknesses for the implementation of family-centered nursing interventions that support parental coping or parenting more generally during a child's burn injury hospitalisation or recovery
